# Evaluation of variants in the selectin genes in age-related macular degeneration

**DOI:** 10.1186/1471-2350-12-58

**Published:** 2011-04-26

**Authors:** Robert F Mullins, Jessica M Skeie, James C Folk, Frances M Solivan-Timpe, Thomas A Oetting, Jian Huang, Kai Wang, Edwin M Stone, John H Fingert

**Affiliations:** 1 Department of Ophthalmology and Visual Sciences, The University of Iowa, 200 Hawkins Drive, Iowa City, IA, 52242, USA; 2 Department of Statistics and Actuarial Science, The University of Iowa, 241 Schaeffer Hall, Iowa City, IA 52242, USA; 3 Department of Biostatistics, The University of Iowa, C22 General Hospital, Iowa City, IA 52242, USA; 4 The Howard Hughes Medical Institute, 200 Hawkins Drive, Iowa City, IA, 52242, USA

## Abstract

**Background:**

Age-related macular degeneration (AMD) is a common disease of the elderly that leads to loss of the central visual field due to atrophic or neovascular events. Evidence from human eyes and animal models suggests an important role for macrophages and endothelial cell activation in the pathogenesis of AMD. We sought to determine whether common ancestral variants in genes encoding the selectin family of proteins are associated with AMD.

**Methods:**

Expression of E-selectin, L-selectin and P-selectin was examined in choroid and retina by quantitative PCR and immunofluorescence. Samples from patients with AMD (n = 341) and controls (n = 400) were genotyped at a total of 34 SNPs in the *SELE*, *SELL *and *SELP *genes. Allele and genotype frequencies at these SNPs were compared between AMD patients and controls as well as between subtypes of AMD (dry, geographic atrophy, and wet) and controls.

**Results:**

High expression of all three selectin genes was observed in the choroid as compared to the retina. Some selectin labeling of retinal microglia, drusen cores and the choroidal vasculature was observed. In the genetic screen of AMD versus controls, no positive associations were observed for *SELE *or *SELL*. One SNP in *SELP *(rs3917751) produced p-values < 0.05 (uncorrected for multiple measures). In the subtype analyses, 6 SNPs (one in *SELE*, two in *SELL*, and three in *SELP*) produced p-values < 0.05. However, when adjusted for multiple measures with a Bonferroni correction, only one SNP in *SELP *(rs3917751) produced a statistically significant p-value (p = 0.0029).

**Conclusions:**

This genetic screen did not detect any SNPs that were highly associated with AMD affection status overall. However, subtype analysis showed that a single SNP located within an intron of *SELP *(rs3917751) is statistically associated with dry AMD in our cohort. Future studies with additional cohorts and functional assays will clarify the biological significance of this discovery. Based on our findings, it is unlikely that common ancestral variants in the other selectin genes (*SELE *and *SELL*) are risk factors for AMD. Finally, it remains possible that sporadic or rare mutations in *SELE*, *SELL*, or *SELP *have a role in the pathogenesis of AMD.

## Background

Age-related macular degeneration (AMD) is a common disease of the elderly that leads to loss of the central visual field due to atrophic or neovascular events. Prevalence rates vary between populations[1], and as many as 64% of individuals over the age of 80 may be affected to some degree[2]. Despite the common prevalence of this disease, the molecular and cellular events that lead to AMD are not well understood.

One observation that has been made in human eyes with AMD, and in some relevant animal models, is that local inflammatory events are associated with the progression of the disease [3,4]. These events include elevated numbers of choroidal leukocytes and/or altered behavior of these cells in eyes with AMD [5-8]. Evidence for a potentially harmful role for monocytes and neutrophils in the etiology of choroidal neovascularization has been provided by animal models of neovascular disease, in which depletion of leukocyte populations has been found to ameliorate laser induced neovascularization [9-11], although the role of macrophages in mouse models of CNV may depend of the modality by which they are depleted and by the age of the mouse leukocytes used in the experiment [12,13].

The collective data indicating that the recruitment of monocytes and neutrophils into the choroid and/or retina occurs during the pathogenesis of AMD suggest that increased leukocyte trafficking occurs in AMD at the level of the choroidal microvasculature. While the process of extravasation is essential in responding to pathogens and maintaining a sterile environment, excessive inflammation can lead to tissue damage in other systems [14,15] Assuming that the mechanisms of leukocyte recruitment to the choroid are similar to those in other tissues, the principal molecules involved in this process are soluble chemokines and cell surface adhesion molecules. Among the latter are integrins, immunoglobulin superfamily members, and the selectins.

The selectins are type I transmembrane proteins characterized by an N-terminal lectin domain, an EGF like domain and a series of complement regulatory domains in the extracellular space. These proteins function in the early phases of leukocyte recruitment by promoting rolling behavior in leukocytes [16]. Similar to other endothelial cell activation molecules, expression of selectins is upregulated by a variety of insults including oxidative injury [17,18] and complement attack [19]--factors widely proposed as central in the pathogenesis of AMD. Unlike other endothelial cell adhesion molecules, selectins act through protein-carbohydrate interactions, binding to carbohydrate epitopes on glycosylated target proteins [20].

In view of the role of inflammatory events and leukocyte recruitment in AMD, we hypothesized that molecules that mediate binding of leukocytes to the endothelium would be involved in the pathogenesis of AMD, and that variations in the genes that encode these adhesion molecules might be associated with the disease. To test this hypothesis, we evaluated the expression of E-selectin, L-selectin and P-selectin in the retina and choroid of aging human donor eyes. We also evaluated 34 SNPs in the *SELE*, *SELL *and *SELP *genes to assess whether ancestral variants in these genes contribute to the risk of AMD.

## Methods

### Expression of selectins

Human donor eyes were obtained from the Iowa Lions Eye Bank within 5 hours of death following informed consent of the next of kin. All experiments were performed in accordance with the tenets of the Declaration of Helsinki. Expression of selectin genes was also assessed in human retina and RPE-choroid using reverse transcriptase PCR and immunohistochemistry. Briefly, RNA was extracted from samples of normal human neural retina (n = 3 donors) and RPE-choroid (n = 3 donors) snap frozen with in 5 hours of death. The three donors were ages 79, 80 and 84 and had no known history of macular disease. Samples were collected immediately temporal to the macula and RNA was isolated using the RNeasy kit according to manufacturer's instructions (Qiagen, Valencia, CA). Reverse transcription was carried out using the RT^2 ^First Strand Kit (SABiosciences, Frederick, MD) and quantitative PCR was performed using the Human Extracellular Matrix & Adhesion Molecules Superarray (SABiosciences, Frederick, MD) according to the manufacturer's instructions. Values for *SELE*, *SELL*, and *SELP *were collected for neural retina and RPE/choroid and were normalized to the values for the housekeeping genes beta actin (*ACTB*) and glyceraldehyde-3-phosphate dehydrogenase (*GAPDH*) using the the ΔΔC_t _method.

Immunohistochemistry was performed on human macular punches without AMD containing retina, RPE, choroid and sclera (n>3). Immunohistochemistry was performed as described previously [21] using antibodies directed against P-selectin (R&D Systems), E-selectin (Santa Cruz) and L-selectin (Santa Cruz). Antibodies were diluted 1:50 to 1:200. Primary antibodies were visualized using Alexa-488-conjugated secondary antibodies (Invitrogen; Carlsbad, CA). For P-selectin, an additional 9 eyes with early AMD, 5 eyes with end-stage wet AMD, and 9 control eyes were also evaluated with immunofluorescence. Sections were counterstained with diamidino-phenol-indole (DAPI) and for some experiments were dual-labeled with *Ulex europaeus *agglutinin-1 (Vector Laboratories, Burlingame, CA) which labels both normal and neovascular EC, as described previously [22].

### Genotyping

The study was approved by the University of Iowa's Institutional Review board and informed consent was obtained from study participants. A cohort of 341 subjects with AMD and 400 control subjects, all from the University of Iowa Department of Ophthalmology and Visual Sciences, were enrolled using standard criteria. For the purposes of this study, AMD was defined as AREDS grade 2 or higher and patients were further categorized with either dry AMD, wet AMD, or geographic atrophy using criteria from previous studies [23]. The control subjects were judged not to have AMD after a complete eye exam. A total of 34 SNPs in the SELE, SELL, and SELP genes were selected using HAPMAP data to maximize the power to detect an association using the UCLA Association Study Design Server online software package http://design.cs.ucla.edu/. The cohorts were genotyped at 7 SNPs within the *SELE *gene, 11 SNPs within the *SELL *gene, and 16 SNPs within the *SELP *gene using a mass spectroscopy-based system (Sequenom, San Diego, CA). Genotyping was conducted using the MassArray platform and iPlex Gold reagents with the manufacturer's protocol by GeneSeek (Lincoln, NE). SNP Genotypes and allele frequencies were compared between AMD patients and controls using Chi Square analysis. For rare variants for which Chi Square test was unsuitable, we utilized the Fisher's test. The Bonferroni correction was used to adjust p-values for multiple measures using a gene-based approach to analyze 3 genes (*SELP*, *SELL*, and *SELE*) and 3 phenotypes (Dry AMD, GA, Wet AMD), which gave an adjusted threshold for significance of 0.05/9 = 0.0056. This gene-based Bonferroni correction was validated by analyzing the contiguous genomic region spanning *SELP*, *SELL*, and *SELE *with the Haploview software package (data not shown). Only two strong linkage disequilibrium blocks were detected in the region (SNPs in *SELL *and *SELE *are in strong linkage disequilibrium with each other) which suggests that our correction with 3 genes may be conservative. Hardy-Weinberg equilibrium (HWE) was estimated by analyzing 1,000,000 simulations using the R statistics software package http://www.r-project.org. An arbitrary threshold for HWE was set at p > 0.001. One SNP (rs6693963) did not meet these criteria and was removed from the analysis.

## Results

### Expression studies

Expression of selectin mRNAs was evaluated using a commercial quantitative PCR array. Both retina and RPE-choroid showed expression of all three selectin genes, but the normalized expression in RPE-choroid was much greater than seen in retina (3.4x, 5.1x, and 50.6x higher for *SELL*, *SELE *and *SELP*, respectively).

**Figure 1:**
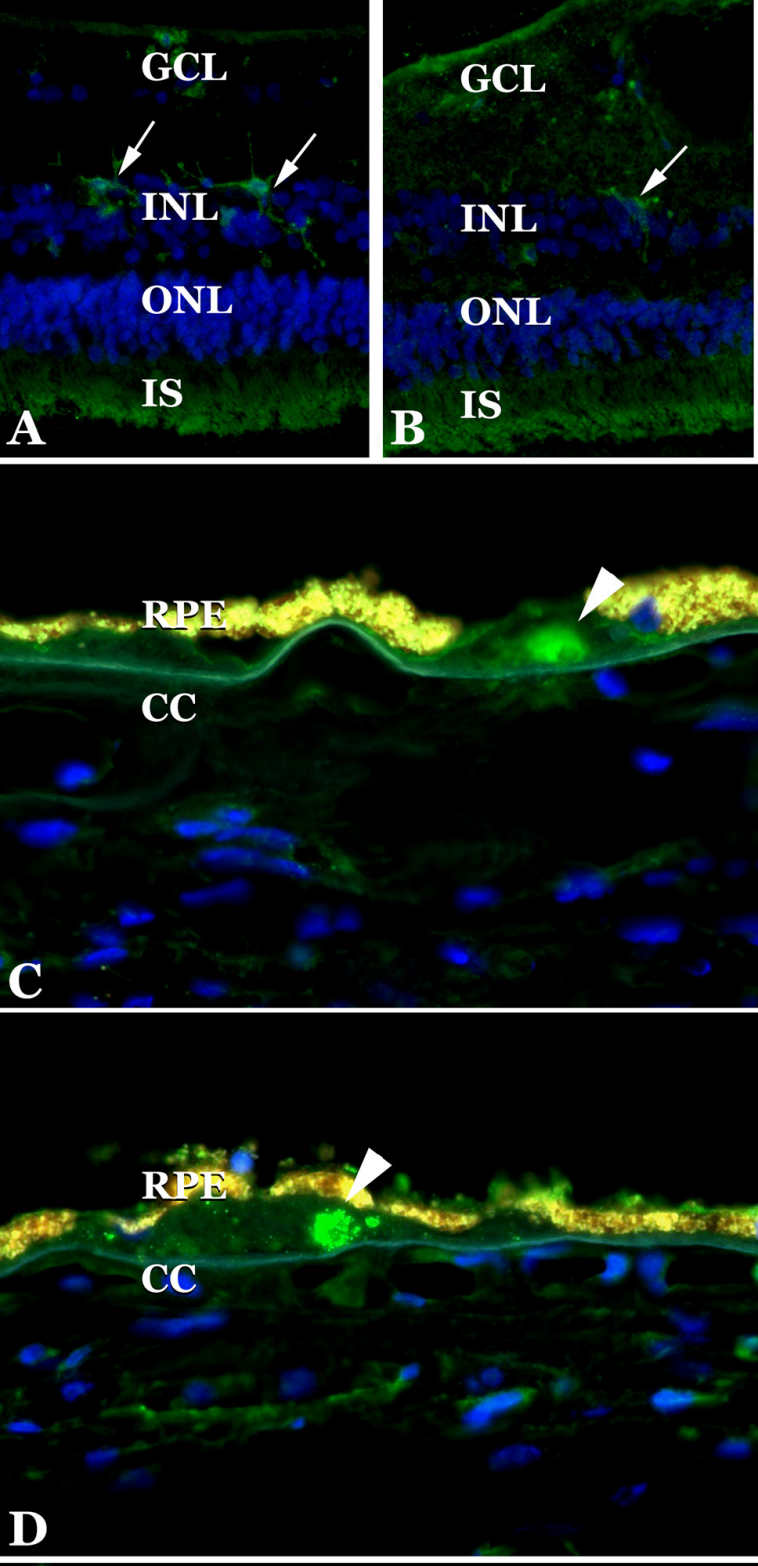
**Expression of E-selectin and L-selectin in retina and choroid**. Antibodies directed. against E-selectin (A, C) and L-selectin (B, D) were found to react with microglia in some retinas (A, B, arrows) as well as the cores of drusen (arrowheads, C, D). Blue fluorescence is due to DAPI staining. Note the bright yellow autofluorescence of the RPE. GCL, ganglion cell layer; INL, inner nuclear layer; ONL, outer nuclear layer; IS, inner segments; RPE, retinal pigment epithelium; CC, choriocapillaris.

**Figure 2:**
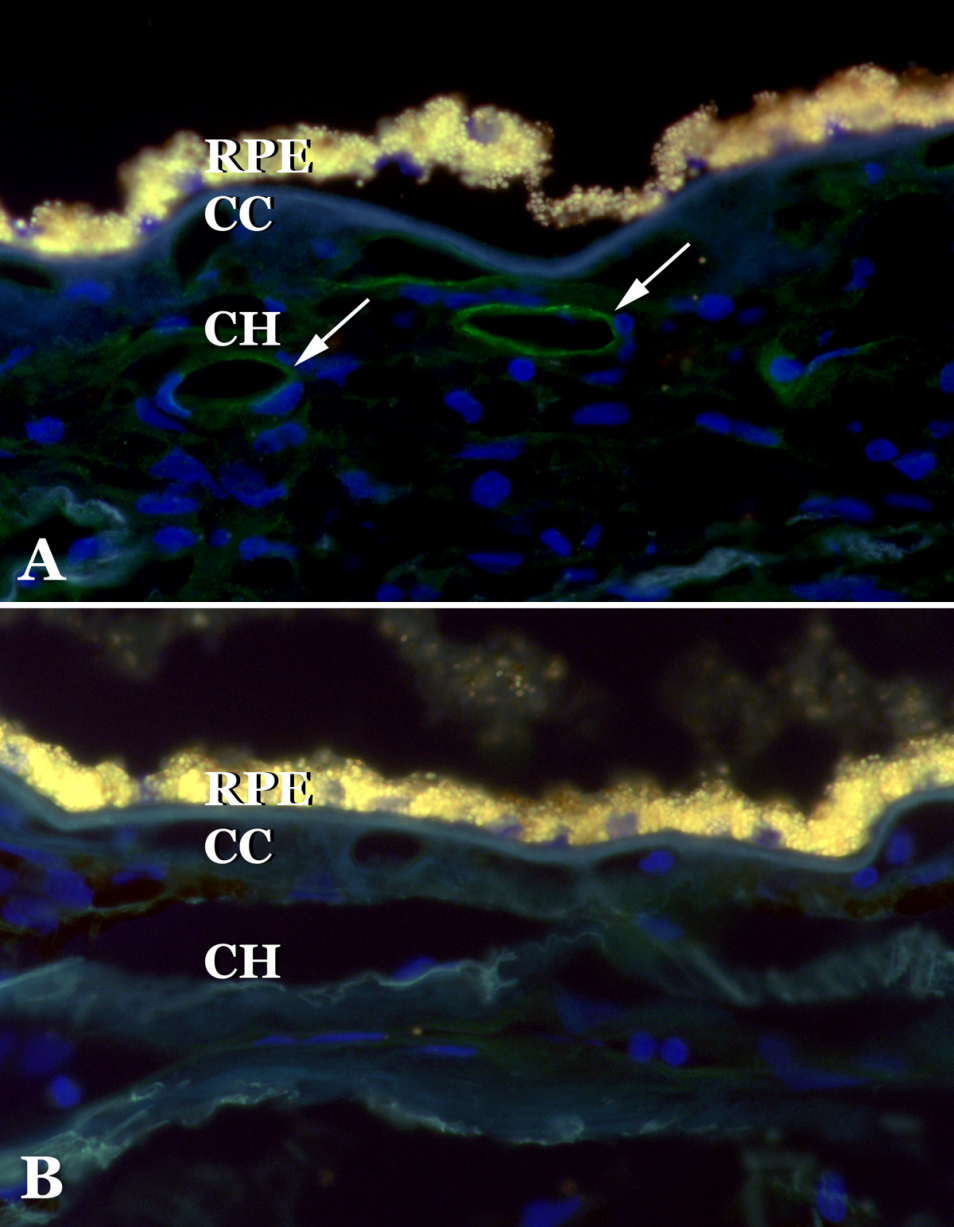
**Localization of P-selectin to choroidal vessels**. The endothelium of large choroidal vascular elements in Sattler's and Haller's layers of the choroid showed immunoreactivity with antibodies directed against P-selectin (A). Modest labeling is observed in the choriocapillaris. Labeling was not observed when the primary antibody was omitted (B). RPE, retinal pigment epithelium; CC, choriocapillaris; CH, outer choroid.

**Figure 3:**
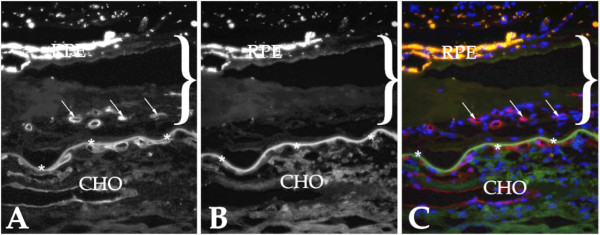
**Evaluation of a human choroidal neovascular membrane with anti-P-selectin antibody**. Sections of affected eyes were dual labeled with UEA-1 lectin, which binds both normal and neovascular endothelial cells (A), and antibodies directed against P-selectin (B). Panel C shows the merged image with DAPI (blue; UEA-I labeling appears red and anti-P-selectin labeling appears green). Labeling of the vasculature with P-selectin antibody was unremarkable. The extent of the neovascular membrane is indicated by the bracket. Arrows indicate endothelial cells within the neovascular complex and asterisks indicate points along Bruch's membrane. RPE, two layer of dystrophic RPE cells above the neovascular membrane; CHO, choroid.

Selectins were localized in sections of human choroid. Overall, as assessed by immunohistochemistry, expression was similar to that seen in other vessel beds and was much weaker than generally observed in the choroid for other adhesion molecules (e.g., ICAM-1 and ICAM-2 [24]). E-selectin was observed on retinal cells we interpreted to be microglia and normal choroidal endothelial cells (Figure 1A), in addition some drusen with core domains [25] showed immunoreactivity (Figure 1C). Drusen cores were also reactive with antibodies directed against L-selectin (Figure 1D), consistent with the observation of other leukocyte antigens associated with these domains[3]. L-selectin also showed some immunoreactivity in cells we interpreted to be microglia (Figure 1B). P-selectin antibodies reacted with the choroidal endothelium of medium and large caliber vessels (Figure 2A). Reactivity was generally not observed in the microvasculature. When present, clusters of platelets were immunoreactive. We also evaluated P-selectin labeling in a series of 18 donor eyes (9 control and 9 early AMD). Labeling intensity varied donor-to-donor, without a clear relationship to disease status. Findings for P-selectin were similar to those described previously in normal and diabetic choroid[26]. Moreover, 5 eyes with neovascular AMD were also assessed with anti-P-selectin antibody. Little or no labeling was detected in the endothelial cells in the neovascular membranes (Figure 3).

### SNP analysis of selectin genes

A cohort of 341 AMD patients and 400 control subjects from Iowa were genotyped at a total of 34 SNPS in the *SELE*, *SELL*, and *SELP *genes. Of the 341 AMD patients, 126 (37%) had dry AMD, 41 (12%) had geographic atrophy, and 174 (51%) had wet AMD. High quality genotypes were obtained at 32 (96%) of the 34 SNPs with an average spacing of 2.5 kb between each SNP and an average call rate of over 97%. However, two SNPs in *SELL *were eliminated from our analysis because one was not polymorphic in our cohort (rs4987382) and the other violated Hardy-Weinberg equilibrium (rs6693963). The allele frequencies and genoytpe frequencies for these 30 SNPs are shown in Table 1. There was no significant difference between the AMD subjects and the control subjects when the allele frequencies and the genotype frequencies of the 15 SNPs in *SELE *and *SELL *were compared (p > 0.05 uncorrected for multiple measures). When the allele frequencies and genotype frequencies of the 16 SNPs in *SELP *were compared between AMD patients and controls, one SNP (rs3917751) produced p-values < 0.05 (uncorrected for multiple measures). However, when adjusted for multiple measures with a Bonferroni correction neither genotype nor allele p-values are statistically significant. The other 15 SNPs in *SELP *produced p-values > 0.05 (Table 1).

**Table 1:** SNPs evaluated in *SELE*, *SELL *and *SELP*.

Gene	rs ID	Affect on encoded protein	Position on chromosome 1	Spacing (bp)	MAF (AMD cohort)	MAF (NL cohort)	MAF (HapMap CEU)	HWE (p-value)	P-values	
									
									Allele frequencies	Genotype frequencies
SELE	rs4786	-	167,958,756		0.23	0.22	0.24	0.39	0.66	0.74

SELE	rs3917438	-	167,960,474	1,718	0.068	0.055	0.05	0.050	0.33	0.27

SELE	rs5368	His468Tyr	167,963,570	3,096	0.099	0.094	0.08	0.39	0.79	0.92

SELE	rs2076059	-	167,965,545	1,975	0.42	0.42	0.44	0.019	>0.99	0.51

SELE	rs3917454	-	167,967,477	1,932	0.030	0.032	0.051	0.63	0.88	0.53

SELE	rs5361	Ser149Arg	167,967,684	207	0.097	0.11	0.092	0.049	0.39	0.54

SELE	rs12408179	-	167,974,751	7,067	0.15	0.17	0.17	0.24	0.66	0.46

										

SELL	rs909628	-	167,927,289		0.090	0.11	0.092	>0.99	0.33	0.51

SELL	rs2298902	-	167,929,703	2,414	0.11	0.11	0.12	0.25	>0.99	0.85

SELL	rs2223286	-	167,932,256	2,553	0.32	0.31	0.27	0.17	0.61	0.48

SELL	rs4987351	-	167,933,979	1,723	0.48	0.50	0.45	0.017	0.49	0.23

SELL	rs2298900	-	167,935,644	1,665	0.33	0.34	0.36	0.080	0.78	0.65

SELL	rs2298899	-	167,936,356	712	0.093	0.083	0.075	0.26	0.52	0.64

SELL	rs4987318	-	167,938,102	1,746	0.23	0.22	0.2	0.34	0.66	0.82

										

SELP	rs3917843	-	167,826,881		0.043	0.041	0.05	>0.99	0.90	0.78

SELP	rs17522707	-	167,829,686	2,805	0.097	0.095	0.058	0.081	0.85	0.29

SELP	rs6136	Thr756Pro	167,830,575	889	0.10	0.11	0.092	0.086	0.55	0.45

SELP	rs1569471	-	167,830,754	179	0.22	0.21	0.18	0.037	0.61	0.11

SELP	rs6133	Val640Leu	167,831,970	1,216	0.12	0.11	0.12	0.075	0.75	0.36

SELP	rs6127	Asp603Asn	167,832,937	967	0.49	0.44	0.48	0.024	0.11	0.20

**SELP**	**rs3917751**	**-**	**167,843,192**	**10,255**	**0.31**	**0.38**	**0.38**	**0.25**	**0.0077**	**0.018**

SELP	rs3917740	-	167,845,890	2,698	0.20	0.21	0.23	0.82	0.70	0.77

SELP	rs3917739	-	167,846,002	112	0.37	0.37	0.33	0.18	0.82	0.72

SELP	rs2235304	-	167,846,377	375	0.051	0.066	0.058	0.10	0.27	0.25

SELP	rs3917734	-	167,847,087	710	0.30	0.29	0.32	0.59	0.73	0.93

SELP	rs6131	Ser331Asn	167,847,509	422	0.16	0.16	0.22	0.58	>0.99	0.62

SELP	rs6125	Val209Met	167,848,941	1,432	0.052	0.050	0.067	0.25	0.90	0.90

SELP	rs3917686	-	167,857,788	8,847	0.11	0.11	0.083	0.13	0.56	0.50

SELP	rs3917682	-	167,858,172	384	0.41	0.40	0.433	0.066	0.75	0.41

SELP	rs3917681	-	167,858,327	155	0.11	0.11	0.125	0.57	0.80	0.86

MAF = Minor allele frequency. HWE = Hardy-Weinberg equilibrium. Reported p-values are uncorrected for multiple measures and those uncorrected p-values < 0.05 are shaded grey. No SNPs produced p-values below the corrected threshold for significance of 0.0042.

Patients were divided into AMD subgroups (dry AMD, wet AMD, and geographic atrophy and further analyzed for associations with the SNPs in *SELE*, *SELL*, and *SELP *(Table 2). When genotype and allele frequencies between each of these AMD subgroups and normal controls were compared, a total of six SNPs (one in *SELE*, two in *SELL*, and three in *SELP*) produced p-values < 0.05. However, after multiple measures corrections, only one SNP in *SELP *(rs3917751) produced a statistically significant p-value of 0.0029.

**Table 2:** Sub-group analysis of SNPs in *SELE*, *SELL *and *SELP*.

Gene	rs ID	Affect on encoded protein	Dry AMD vs. Normals Fisher's extact test		GA vs. Normals Fisher's extact test		Wet AMD vs. Normals Fisher's extact test	
			
			Allele Frequency p-value	Genotype Frequency p-value	Allele Frequency p-value	Genotype Frequency p-value	Allele Frequency p-value	Genotype Frequency p-value
*SELE*	rs4786	-	0.38	0.54	0.087	0.21	0.44	0.56

*SELE*	rs3917438	-	0.75	0.73	0.43	0.70	**0.028**	**0.039**

*SELE*	rs5368	His468Tyr	0.81	0.35	0.42	0.20	0.52	0.75

*SELE*	rs2076059	-	0.65	0.62	0.46	0.67	0.42	0.28

*SELE*	rs3917454	-	>0.99	>0.99	0.74	0.73	>0.99	0.29

*SELE*	rs5361	Ser149Arg	0.91	0.61	0.35	0.45	0.47	0.71

*SELE*	rs12408179	-	0.68	0.92	0.74	0.71	0.36	0.13

								

*SELL*	rs909628	-	>0.99	0.93	0.12	0.35	0.34	0.65

*SELL*	rs2298902	-	0.34	0.12	0.70	>0.99	0.30	0.53

*SELL*	rs2223286	-	0.88	0.71	0.90	0.97	0.44	0.43

*SELL*	rs4987351	-	0.61	0.30	0.10	0.28	>0.99	0.37

*SELL*	rs2298900	-	0.88	0.73	**0.036**	0.096	0.21	0.28

*SELL*	rs2298899	-	0.31	0.45	0.19	0.25	0.42	0.32

*SELL*	rs4987318	-	0.30	0.54	**0.045**	0.13	0.49	0.60

								

*SELP*	rs3917843	-	0.22	0.21	0.762	0.76	0.74	0.20

*SELP*	rs17522707	-	0.62	0.72	0.42	0.92	0.83	0.26

*SELP*	rs6136	Thr756Pro	**0.030**	0.090	0.71	0.67	0.61	0.15

*SELP*	rs1569471	-	0.66	0.50	0.77	0.60	0.53	0.14

*SELP*	rs6133	Val640Leu	0.91	0.45	>0.99	0.63	0.49	0.62

*SELP*	rs6127	Asp603Asn	**0.023**	**0.041**	0.24	0.50	0.84	0.94

*SELP*	**rs3917751**	-	0.0029*	**0.0066**	0.093	0.012	0.31	0.14

*SELP*	rs3917740	-	0.65	0.52	0.19	0.31	0.69	0.90

*SELP*	rs3917739	-	0.48	0.45	0.54	0.55	0.20	0.31

*SELP*	rs2235304	-	0.88	0.88	0.078	0.069	0.28	0.26

*SELP*	rs3917734	-	0.94	0.99	0.70	0.91	0.39	0.65

*SELP*	rs6131	Ser331Asn	0.56	0.35	0.75	0.54	0.79	0.94

*SELP*	rs6125	Val209Met	>0.99	>0.99	0.59	0.58	0.77	0.76

*SELP*	rs3917686	-	0.30	0.39	0.36	0.28	0.76	0.81

*SELP*	rs3917682	-	0.56	0.81	0.23	0.10	0.60	0.36

*SELP*	rs3917681	-	0.57	0.72	0.25	0.092	0.76	0.91

Reported p-values are uncorrected for multiple measures and those uncorrected p-values < 0.05 are indicated by bold text. One SNPs in *SELP *(rs3917751) that is indicated by bold text and an asterisk produced a p-value below the corrected threshold of 0.0042.

## Discussion

Endothelial cell activation is a term that refers broadly to the set of responses that the endothelium undergoes to promote adhesion and extravasation of leukocytes from the lumen to the extravascular space of a tissue. Among the molecular changes that characterize endothelial cell activation is upregulation of adhesion molecules on the luminal surface of the endothelium (e.g., E-selectin) and/or mobilization of intracellular stores of adhesion molecules and redistribution to the cell surface (e.g., P-selectin).

We consider endothelial cell activation molecules to be attractive targets for AMD pathogenesis for the several reasons. First, leukocytes have been noted to be elevated in human eyes with AMD[5-8], as well as associated with drusen. The presence of dispersed MHC class II antigens in drusen may support a causative rather than beneficial role for choroidal leukocytes in drusen pathophysiology[3,27]. Second, depletion of monocytes [9,10] and neutrophils [11] has been found to reduce the severity of neovascularization in animal models of CNV. Third, blood derived macrophages are components of neovascular membranes in human [28,29] and murine [30] eyes with CNV. Moreover, targeted deletion of the Ig superfamily gene *Icam1 *appears protective against experimental CNV in mouse[31], indicating a direct link between endothelial cell-leukocyte interactions and neovascularization and endothelial cell adhesion molecules, including E-selectin, ICAM-1 and ICAM-2 have been observed in human neovascular membranes [24,32].

We evaluated expression and a number of genetic variants in the selectin genes. All three of the selectin gene products were detected at the mRNA and protein level. Both SELE and SELL were localized to cells in the inner retina with a dendritic morphology, interpreted to be retinal microglia, cells that alter their phenotype in eyes with AMD [33]. E-selectin and P-selectin were further identified on some choroidal endothelial cells, where they can bind carbohydrate epitopes on circulating leukocytes.

In light of the evidence linking endothelial cell activation molecules and AMD, we hypothesized that variants in the genes for these molecules might be skewed in AMD patients. The functional impact of variants in these genes could be difficult to predict: since the helpful or harmful roles of monocytes in AMD is somewhat controversial[34], it is plausible that alleles that confer either a gain or loss of function could be involved in AMD pathophysiology.

Our focused association study was designed to search for ancestral mutations in the selectin genes (*SELE*, *SELL*, and *SELP*) that might be common risk factors for AMD. This genetic screen did not detect any SNPs that were highly associated with AMD affection status. Our results for the *SELE *S149R SNP (rs5361) were similar to those of Bojanowski et al., [35] who previously genotyped this SNP in 88 AMD and 110 control samples and found no association with AMD. However, one SNP located within an intron of the P-selectin gene (rs3917751) showed a modest association with AMD overall, but given the number of SNPs and genes evaluated, this observation is not statistically significant.

When subtypes of AMD were individually analyzed, one *SELP *SNP (rs3917751) produced a p-value of 0.0029 that was statistically significant after correction for multiple measures. The SNP rs3917751 is located within an intron of *SELP *and its biological significance is unclear. Further study with additional cohorts and functional assays will likely clarify the potential role of rs3917751 and *SELP *in the pathogenesis of dry AMD.

Overall, the association study detected evidence of one ancestral risk allele for dry AMD in *SELP *(rs3917751). No associations were detected with studies of AMD overall or with wet AMD or geographic atrophy. However, as association studies are unable to identify non-ancestral risk alleles, it remains possible that sporadic or rare mutations in *SELE*, *SELL*, or *SELP *have a role in the pathogenesis of AMD.

## Conclusions

This genetic screen did not detect any SNPs that were highly associated with AMD affection status overall. However, subtype analysis showed that a single SNP located within an intron of *SELP *(rs3917751) is statistically associated with dry AMD in our cohort. Future studies with additional cohorts and functional assays will clarify the biological significance of this discovery. Based on our findings, it is unlikely that common ancestral variants in the other selectin genes (*SELE *and *SELL*) are risk factors for AMD. Finally, it remains possible that sporadic or rare mutations in *SELE*, *SELL*, or *SELP *have a role in the pathogenesis of AMD.

## Competing interests

The authors declare that they have no competing interests.

## Authors' contributions

RFM contributed to the design of the study, directed and performed immunofluorescence studies, contributed to analysis of data, and participated in writing the manuscript. JMS performed immunofluorescence studies, contributed to analysis of the data, and participated in writing the manuscript. JCF examined and diagnosed patients and enrolled study subjects. FST performed immunofluorescence studies and analyzed and interpreted the genotype data. TAO examined and diagnosed patients and enrolled study subjects. JH participated in study design, directed statistical analyses, and participated in data interpretation. KW also participated in study design, directed statistical analyses, and participated in data interpretation. EMS examined and diagnosed patients and enrolled study subjects, contributed to analysis of the data, and participated in editing the manuscript. JHF participated in the study design, oversaw the genotyping studies, participated in the statistical analyses, and participated in writing the manuscript. All authors read and approved the final manuscript.

## Pre-publication history

The pre-publication history for this paper can be accessed here:

http://www.biomedcentral.com/1471-2350/12/58/prepub
